# Leaf economics and slow-fast adaptation across the geographic range of *Arabidopsis thaliana*

**DOI:** 10.1038/s41598-019-46878-2

**Published:** 2019-07-24

**Authors:** Kevin Sartori, François Vasseur, Cyrille Violle, Etienne Baron, Marianne Gerard, Nick Rowe, Oscar Ayala-Garay, Ananda Christophe, Laura Garcia de Jalón, Diane Masclef, Erwan Harscouet, Maria del Rey Granado, Agathe Chassagneux, Elena Kazakou, Denis Vile

**Affiliations:** 1 Univ Montpellier, CNRS, EPHE, IRD, Univ Paul Valéry Montpellier 3, Montpellier, France; 2 Univ Montpellier, INRA, Montpellier SupAgro, LEPSE, Montpellier, France; 3 Univ Montpellier, CIRAD, CNRS, INRA, IRD, Montpellier, France; 4 Programa de Recursos Genéticos y Productividad (RGP)-Fisiología Vegetal, Colegio de Postgraduados, 56230 Texcoco, Mexico; 5 Office National de la Chasse et de la Faune sauvage, DRE Unité, Ongulés sauvages, Birieux, France; 6 Univ Montpellier, INRA, Montpellier SupAgro, Montpellier, France

**Keywords:** Ecological genetics, Natural variation in plants, Plant ecology, Plant evolution

## Abstract

Life history strategies of most organisms are constrained by resource allocation patterns that follow a ‘slow-fast continuum’. It opposes slow growing and long-lived organisms with late investment in reproduction to those that grow faster, have earlier and larger reproductive effort and a short longevity. In plants, the Leaf Economics Spectrum (LES) depicts a leaf-level trade-off between the rate of carbon assimilation and leaf lifespan, as stressed in functional ecology from interspecific comparative studies. However, it is still unclear how the LES is connected to the slow-fast syndrome. Interspecific comparisons also impede a deep exploration of the linkage between LES variation and adaptation to climate. Here, we measured growth, morpho-physiological and life-history traits, at both the leaf and whole-plant levels, in 378 natural accessions of *Arabidopsis thaliana*. We found that the LES is tightly linked to variation in whole-plant functioning, and aligns with the slow-fast continuum. A genetic analysis further suggested that phenotypic differentiation results from the selection of different slow-fast strategies in contrasted climates. Slow growing and long-lived plants were preferentially found in cold and arid habitats while fast growing and short-lived ones in more favorable habitats. Our findings shed light on the role of the slow-fast continuum for plant adaptation to climate. More broadly, they encourage future studies to bridge functional ecology, genetics and evolutionary biology to improve our understanding of plant adaptation to environmental changes.

## Introduction

Investigations of the genetic and phenotypic differentiation of plant lineages along environmental gradients is crucial for the understanding of plant evolutionary responses to current and future climate variations. As plants cannot simultaneously optimize competing eco-physiological functions, an important question is how plant adaptation occurs under the influence of major trade-offs between traits.

The slow-fast continuum is a pervasive trade-off between resource allocation to growth, reproduction and survival, spread across the tree of life^1^. The slow end of this continuum is characterized by slow growing, long-lived species and low reproductive output, while species at the fast end reach reproductive maturity faster and produce more offsprings. In plants, the leaf economics spectrum (LES hereafter)^2–4^ is thought to reflect the physiological basis of the slow-fast continuum^4^. The LES arrays plant species along a continuum of leaf trait syndromes going from short-lived leaves with fast metabolism to the reverse syndrome^3^. Core LES traits include leaf dry mass per area (LMA), leaf lifespan (LLS) and net photosynthetic rate per mass unit (*A*_mass_)^3,5–7^. LES traits are widely used in comparative ecology to infer whole-plant ecological strategies^4,8–12^. However, the extent to which leaf-level resource economics reflects whole-plant physiology, performance, and ultimately fitness, is still under debate^13^. Many processes can lead to a mismatch between LES and whole-plant functioning^14^, including the impact of self-shading among leaves and resource allocation patterns, such as carbon investment in non-photosynthetic tissues^15,16^. To gain insights into the robustness of the slow-fast continuum at different organizational levels, we need to examine how LES traits scale up to plant level resource-use strategies, life history and performance. However, it remains difficult to compare individual performance across species with different growth forms, phenology and dispersal strategies since cross-species comparisons impede a clear linkage between physiological and adaptive trade-offs^17–19^.

The LES has been associated with differences in the ability of plants to adapt to more or less harsh environmental conditions^4,12,20,21^: species displaying high photosynthetic, respiration and growth rates, are short-lived, with thin and nitrogen-rich leaves are preferentially found in nutrient-rich and/or growth-suitable climatic conditions. Those species are qualified as acquisitive species in contrast to conservative ones that exhibit the opposite set of traits. Despite these observations, functional ecology has no tools to test for adaptation, and empirical evidences of the adaptive value of being at one end or the other of the continuum in a given environment remain scarce (see ref.^22^ for a review). Furthermore, sampling procedure in field observation studies often impedes to disentangle the effects of plasticity vs. genetic differentiation on the emergence of the LES^23^. Thus, comparative studies looking for plant adaptation are at best incomplete^22–24^, and the role of selection in shaping the LES and driving adaptation to diverse environments is hardly understood. To fill this gap, intraspecific studies are encouraged since they can take benefit from tools developed in population ecology and genetics^22,25,26^. The LES has started to be analyzed at the intraspecific level, with contrasting findings depending on the studied organism and type of study^23,27–32^. LES relationships appeared consistent with cross-species ones when using species with broad environmental niche spectra^31–33^ and/or broad phenotypic variability^23^, but inconsistent when using species with narrow phenotypic (and genetic) diversity^34^. Genetic differentiation of LES strategies has been demonstrated among populations of *Helianthus anomalus* along a 400 km rainfall gradient^35^. However, the question whether LES diversifies because of adaptation to climate among lineages spanning large geographic distribution remains open. Overall, we still miss a comprehensive understanding of within-species LES variation and the subsequent insights they can provide to well-described interspecific patterns from an evolutionary perspective.

Within-species genetic and phenotypic diversity is driven by natural selection, genetic drift, mutation and migration. The measure of F_ST_ statistics among populations, i.e. a comparison of genetic or phenotypic differences among populations, is commonly used to distinguish neutral *versus* adaptive processes. At the genetic level, neutral loci are characterized by low F_ST_ values, *i.e*. low between-population differentiation due to the combined effect of migration, mutation and drift, while high F_ST_ values reflect genetic divergence as a consequence of directional selection in contrasted environments. From a phenotypic standpoint, adaptive divergence relies on the Q_ST_ statistics, which was developed, by analogy to F_ST_, to evaluate the population structure for quantitative traits. Q_ST_ values above neutral F_ST_ are interpreted as a signature of diversifying selection on the underlying trait. For instance, Q_ST_-F_ST_ comparisons have been successfully used in *Campanula rotundifolia, Arrhenatherum elatius, Quercus oleoides* and *Arabidopsis thaliana* to investigate the role of selection in the diversification of life-history traits, growth strategies and drought resistance among lineages at both local and global scales^36–39^. This method is expected to be particularly powerful in model species where modern genomics have given access to high-throughput genotyping^40,41^. By comparing genetic and phenotypic differentiation between populations or lineages, Q_ST_-F_ST_ provides a powerful tool to infer adaptation in polygenic quantitative traits such as LES traits^41^.

The species *Arabidopsis thaliana* has been widely used in molecular biology, cell biology and quantitative genetics. Thanks to the efforts to characterize the genetic diversity in this species^42–45^, it is also a model in population dynamics^46^ and evolutionary ecology^47^. For instance, the genetic determinism of *A. thaliana* life history has been extensively studied, notably with the discovery of genes that control major developmental transitions such as flowering time (see ref.^48^ for a review). Allelic variation in these genes appears to be adaptive to climatic and altitudinal gradients^49^. A recent study supports the hypothetic link between life history variation and the LES in *A. thaliana*, highlighted by strong genetic correlations between these traits^28^. However, this analysis was performed on recombinant inbred lines used for genetic mapping. Made of artificial crosses, they preclude examining the relationships between LES and the natural environment. Interestingly, *A. thaliana* has recently gained a renewed interest in functional ecology and biogeography^50–52^, notably due to the large panel of natural accessions that have been collected from contrasting climates, and genotyped at high density (*e.g*.^42–45^). As genetic data in *A. thaliana* allow an unprecedented large-scale analysis of genetic variation among populations and lineages, this species is promising to investigate the extent of intraspecific diversity and its role for adaptation to contrasted climates.

In this study, we explored the evolutionary bases of intraspecific leaf and plant trait variation using a pan-European collection of 378 natural *A. thaliana* accessions from the RegMap panel^44^. Specifically, we investigated whether plant adaptation to various climates is associated with genetic differentiation along the LES and the slow-fast continuum. To test this hypothesis, we first examined how the LES shapes phenotypic diversity across contrasted genotypes of *A. thaliana*, and tested whether LES traits scale up to plant level resource-use strategies and life history. Next, we took benefit from the large genomic information available in *A. thaliana* to evaluate to what extend phenotypic differences among lineages are attributable to adaptive processes such as adaptation to contrasted climates using Q_ST_-F_ST_ comparisons.

## Results

### Geographic clustering of *A. thaliana* lineages

**Figure 1:**
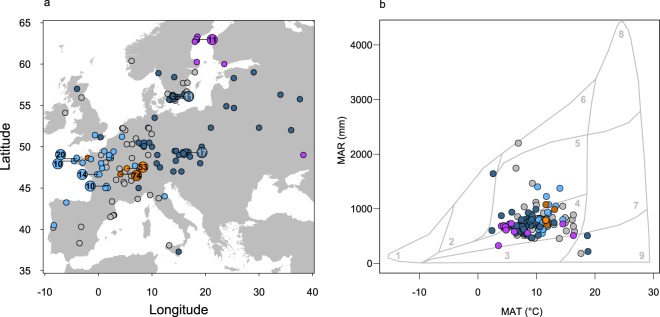
Location and climatic conditions of the genotype collecting sites. (**a**) Distribution of the 378 natural genotypes used in this study. The small points represent the collecting sites of genotypes and bigger points give the number of collecting sites overlapped at these positions. The colors represent the five genetic groups: Admixed (grey), French 1 (brown), French 2 (orange), Swedish (purple), Central Europe (dark blue), Western Europe (light blue). (**b**) Mean annual rainfall (MAR) and mean annual temperature (MAT) for the sites where genotypes were collected, in relation to major biome types of the world following Whittaker’s classification. 1–9: Tundra, Boreal forest, Temperate Grassland Desert, Woodland Shrubland, Temperate Forest, Temperate Rain Forest, Tropical Forest Savana, Tropical Rain Forest, and Desert.

Following the Whittaker’s biome classification method, two climatic variables, mean annual rainfall (MAR) and mean annual temperature (MAT), were used for study site classification into the major biome types. The range of biomes experienced by the sampled genotypes covers woodlands-shrublands and the less humid part of temperate forests, with a few extremes in boreal forests and deserts like environments (Fig. 1b). Using the 250 K SNPs data available from Horton *et al*.^44^, we performed a genetic clustering of the genotype set, which revealed the presence of five genetic lineages. These lineages were moderately differentiated (mean F_ST_ = 0.11), geographically (Fig. 1a) as well as in the Whittaker’s biome classification (Fig. 1b). The analysis revealed the existence of two genetic groups exclusively located in France in our sample (French 1 and French 2 hereafter) of 76 and 49 genotypes, respectively. Among the 20 genotypes of the third group, seven were defined as North Swedish in the 1001 genomes dataset^45^. Consistently, the 20 “Swedish” genotypes, although not all in Sweden (Fig. 1a), were mainly located in cold environments and woodland-shrubland in Whittaker’s classification (Fig. 1b). We considered the 76 genotypes from group 4 as “Central European” (Fig. 1a), typically living at intermediate temperatures and rainfall (Fig. 1b). 83 genotypes composed the group 5, all located in Western Europe (Fig. 1a), in a range of relatively warm environments with intermediate rainfall.

### Leaf economics of *A. thaliana*

**Figure 2:**
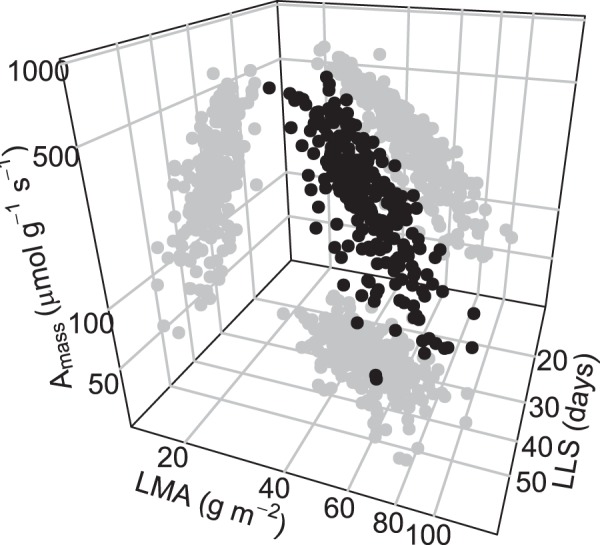
The leaf economics spectrum in *A. thaliana*. Three-way relationships among the main leaf economics traits: A_mass_, mass based assimilation rate (*µ*mol CO_2_ g^−1^ s^−1^); LMA, leaf mass per area (g m^−2^); LLS, leaf lifespan (days). Each point represents a different genotype.

Assimilation rate (*A*_mass_) was the most variable trait among the leaf economics traits in our dataset (15-fold; from 40.9 to 608.9 µmol g^−1^ s^−1^) while leaf mass per area (LMA) and leaf lifespan (LLS) varied 5 and 3.5 fold (from 18.7 to 101 g m^−2^, and from 15 to 53.5 days), respectively. In regard to interspecific meta-analyses, variation in *A*_mass_ was wide (from 5 to 660 µmol g^−1^ s^−1^ ^3^) and was relatively weak for LMA (from 14 to 1,500 g m^−2^ ^3^) and for LLS (from 0.9 to 288 months^3^). Pairwise regression revealed strong correlations between traits, independently of the genetic structure of the sample (*r*²_*A*mass-LLS_ = 0.32, *r*²_*A*mass-LMA_ = 0.73, *r*²_LMA-LLS_ = 0.38, all p-value < 0.01). The 3-dimension space shaped by traits covariations in *A. thaliana* was comparable to the interspecific spectrum previously observed^3^: genotypes are ranked from low *A*_mass_ and high LMA and LLS, toward high *A*_mass_ and low LMA and LLS (Fig. 2). A principal component analysis (PCA) showed that 78% of the covariation between these three traits was explained by a single Principal Component (PC1; Fig. S1a). Hereafter, we assigned a position along the LES for each genotype according to its score on PC1. *A*_mass_ was highly negatively correlated with PC1 (*r* = −0.90) while LMA and LLS were positively correlated with PC1 (*r* = 0.91 and 0.79, respectively). Thus, high and low PC1 values are representative of genotypes located at the conservative and acquisitive side of the LES, respectively.

### From the Leaf Economics Spectrum to the plant slow-fast continuum

**Figure 3:**
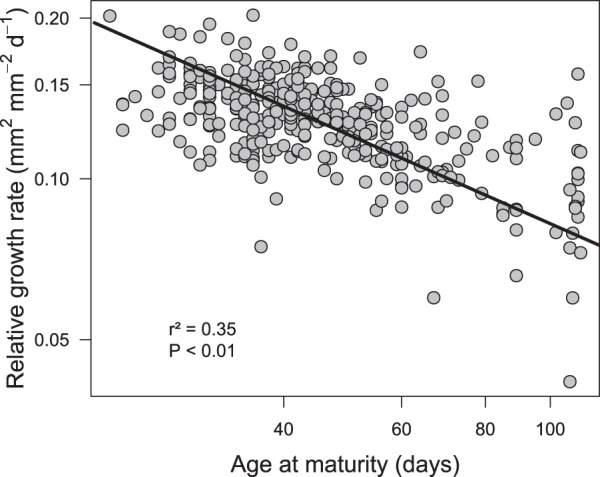
The slow-fast continuum in *A. thaliana*. Relationship between relative growth rate (mm^2^ mm^−2^ d^−1^) and age at maturity (days). Each point represents a different genotype. The solid line represents the output of a linear model, the slopes do not significantly differ while taking into account the genetic relatedness of genotypes or not.

**Figure 4:**
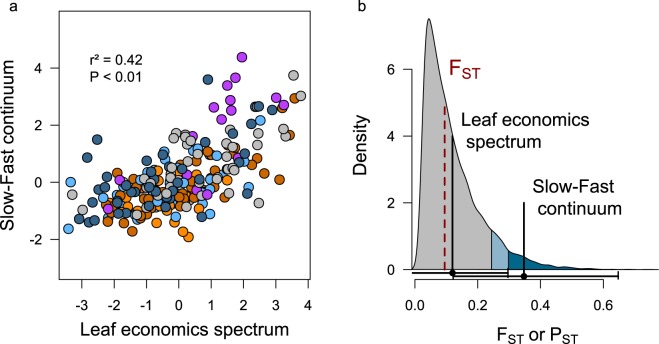
The Leaf Economics Spectrum and the slow-fast continuum covariate in *A. Thaliana* and are differentiated among populations. (**a**) Correlation between the position of each genotype along the leaf economics spectrum and the slow-fast continuum. Colors represent the five genetic groups (see Fig. 1). (**b**) Phenotypic differentiation (P_ST_, black vertical lines) and their confidence intervals (horizontal brackets) relatively to the distribution of genetic differentiation (F_ST_), its median (red dashed line), 90^th^ quantile (light blue) and 95^th^ quantile (dark blue).

Trait measurements at the plant level revealed that assimilation rate was again the most variable trait with a 68-fold variation (from 8.4 to 578.1 µmol g^−1^ s^−1^), while plant mass per area and age of maturity (AM) both varied 5 fold (from 17.7 to 85.4 g m^−2^ and 22 to 111 d, respectively). Standardized major axis (SMA) regressions between traits measured at the leaf and plant levels were all significant. Leaf and whole plant-level LMA were highly correlated (*r* = 0.89; P < 0.001; Fig. S1e) and the slope was close to, but significantly different from 1 (95% Confidence Interval slope = [1.07, 1.18]), as well as for leaf-level and whole plant-level net assimilation rate (*r* = 0.78; P < 0.001; Fig. S1f, 95% CI slope = [0.78, 0.9]). Similarly, LLS and AM were significantly correlated with a slope below 1 (slope = 0.68 [0.55; 0.65], *r* = 0.67, P < 0.001; Fig. S1g). A single principal component explained 86% of the trait covariation at the plant level (Fig. S1b) and was highly correlated with PC1 at the leaf level (*r* = 0.87, P < 0.001; Fig. S1h). Furthermore, life history and performance at the plant level co-varied in parallel with this leaf level phenotypic variation. Relative growth rate (RGR) and age at maturity (AM) were negatively correlated (Fig. 3) in our dataset, consistently with the slow-fast continuum pattern: fast growing genotypes reproduced earlier than slow growing ones. As for the LES, we performed a PCA with slow-fast traits and we assigned a position along the continuum for each genotype according to its score on PC1. The positions of the genotypes along the leaf economics spectrum and the slow-fast continuum were tightly correlated (Fig. 4a) and the slope did not differ significantly when taking the kinship matrix of the genotypes as a covariate. In other words, slow growing and late reproducing genotypes have low leaf carbon assimilation rate and long leaf lifespan, whereas fast growing and early reproducing genotypes have high leaf assimilation rate and short leaf lifespan.

### Adaptation cues of the slow-fast continuum

Consistently with the original description of the RegMap genotypes^44^, the genetic differentiation among *A. thaliana* populations was substantial. Pairwise comparisons based on the whole genetic data indicated a strong genetic divergence between French 2 and the other groups (F_ST_ > 0.19). French 1 group was genetically closer to Western European group (F_ST_ = 0.13) than French 2 group (F_ST_ = 0.20). Interestingly, North Swedish lines showed strong phenotypic and genetic differentiation with other lineages (Fig. S4). We evaluated the significance of the differentiation of the slow-fast strategies performing Q_ST_-F_ST_ comparisons for both RGR and AM. Heritability was high for both traits (*h*²_AM_ > 0.95, *h*²_RGR_ ~ 0.64). P_ST_ is a good estimator of Q_ST_ when trait heritability (*h*²) is high and the phenotypic between-populations component of variance genetically determined (c) is close to *h*² (see the Methods section). For the null hypothesis (H0: c = *h*² = 1), P_ST_ values were high and significantly higher than the neutral F_ST_ for both traits (P_ST AM_ ~ 0.35, P_ST RGR_ ~ 0.3; see Table 1). However, further analyses revealed that the estimation of P_ST_ were highly sensitive to the ratio between the among-population (c) and overall heritability (*h*²) of the trait. On the other hand, we showed that all LES traits, exhibited modest and non-significant P_ST_ values (Table 1) under the null assumption. Finally, we performed a P_ST_ analysis on PCA scores for the two set of traits. Despite a lower heritability (h²_SFC_ ~ 0.59, h²_LES_ >0.95), the slow-fast continuum P_ST_ was high and significant compared to the leaf economics spectrum P_ST_ (Fig. 4b) (P_ST SFC_ ~ 0.35, critical c/h² ~ 0.35; P_ST LES_ ~ 0.12), although both were highly sensitive to c/*h*². This suggests that slow-fast traits behaved like outlier variants that diverged among lineages due to the effect of diversifying selection. Our data do not support the same hypothesis for LES traits, despite a tight coordination with slow-fast traits.

**Table 1:** The phenotypic differentiation of *A. thaliana* regarding slow-fast and LES traits is genetically determined and structured among regional populations.

	LES	SFC	A_mass_	LLS	LMA	AM	RGR
*h*²	0.99	0.59	0.70	0.66	0.88	0.99	0.64
P_ST_	0.12	0.35	0.12	0.02	0.19	0.35	0.3
CI 95%	[−0.02;0.28]	[0.07;0.7]	[−0.01;0.29]	[−0.05;0.09]	[0.01;0.42]	[0.07;0.71]	[0.03;0.62]
Critical c/h²	NA	1.25	NA	NA	NA	NA	1.49

Abbreviations: *h*², narrow-sense heritability; P_ST_, phenotypic differentiation statistic; CI, confidence interval; c, among-population heritability.

### Climatic drivers of *A. thaliana* phenotypes

**Figure 5:**
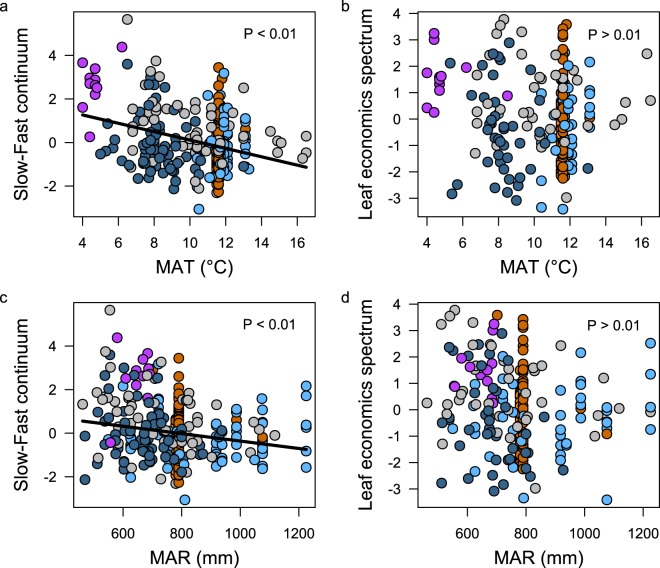
Slow-fast continuum, Leaf Economics Spectrum, and their relationships with climate. Position of genotypes along the slow-fast continuum as a function of (**a**) mean annual temperature (MAT) and (**b**) mean annual rainfall (MAR) and position of genotypes along the Leaf Economics Spectrum as a function of (**c**) MAT and (**d**) MAR. Linear regressions are represented by black lines when significant.

**Figure 6:**
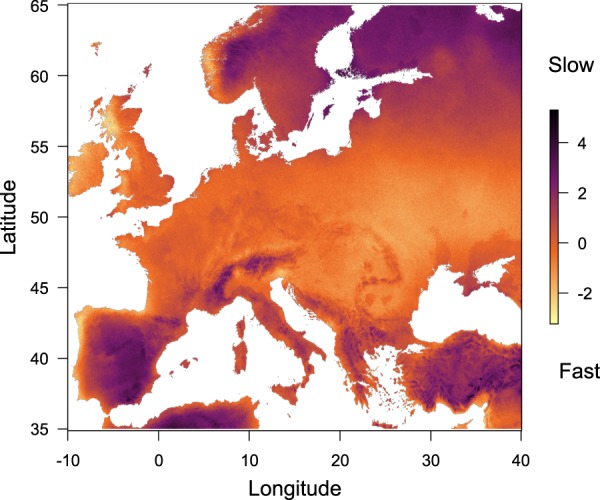
Slow strategy is favored in Mediterranean and Scandinavian regions while fast strategy is selected in the center of *A. thaliana* distribution. Map representing the prediction of slow-fast strategies based on climatic data: darker regions endure combination of climatic variables associated with slower strategies as opposed to faster strategies in lighter regions.

We investigated whether annual rainfall and temperature, two key climatic variables in plant functional biogeography, explain the position of the genotypes along the slow-fast continuum and leaf economics spectrum axes. We extracted 19 climatic variables at the collecting sites of the genotypes on CHELSA website (www.chelsa-climate.org/) related to temperature and precipitation, their temporal variability and extremes. Consistently with the population structure analysis, only the slow-fast continuum axis was significantly correlated with MAR (*r* = −0.18, P < 0.01) and MAT (*r* = −0.16, P < 0.01) (Fig. 5). The correlation with the 17 other CHELSA variables revealed the same pattern: the slow-fast continuum axis and the related traits were more often significantly correlated with climatic variables (Table S1). Therefore, we investigated whether climatic variables at the collecting sites of the genotypes can predict the *A. thaliana* slow-fast strategies. Firstly, stepwise regressions revealed that position on the slow-fast continuum was best predicted by a subset of climatic variables, including the mean annual temperature and rainfall, and variables related to temperature variance and extremes throughout the year. We evaluated the accuracy of the model using a repeated cross-validation method, which revealed that phenotypes were more accurately predicted by the reduced model (*r*² ~ 0.26) than the formal model (*r*² ~ 0.20). Extrapolating prediction of phenotypes across Europe from climate variables, we showed that slow strategies, characterized by slow growth and late reproduction, were favored in North Europe and Central East of Spain and in the highest European reliefs (Fig. 6). In addition, fast strategies characterized by fast growth and early reproduction were found in Central Europe and near the coasts.

## Discussion

The comparison of multiple species based on a few traits is the historical approach of functional ecology^53^. While fruitful^12^, such an approach impedes a deeper investigation of how evolutionary forces and trade-offs operate together to shape the observable phenotypic diversity^24,26^. Notably, several trait-trait covariations have been discussed in functional ecology in the light of trade-off theories. One of the most prominent phenotypic pattern discussed in the last decades, the so-called Leaf Economics Spectrum (LES), is thought to reflect a trade-off between metabolic rate and lifespan at the leaf level^3,5,54,55^. Plant species that exhibit long-lived leaves have been referred as resource conservative species. They optimize long-term carbon gain and extended nutrient residence time, as well as nutrient use efficiency^56^. By contrast species with short-lived leaves sacrifice nutrient retention to maximize the rate of carbon fixation. The LES is expected to reflect an adaptive trade-off between fast and slow growth strategies across plant species^4^. Two assumptions underline this assertion: (i) the negative correlation between leaf photosynthetic rate and leaf lifespan is translated into a negative correlation between plant growth rate and the duration of the life cycle, (ii) particular combinations of slow-fast traits are selected in different environments. Both assumptions are difficult to test at the interspecific level. This has generated a living debate about the evolutionary causes of the LES^22,28,57–60^. Taking benefit from a large collection of sequenced genotypes in a model species, our results show that LES traits are correlated with slow-fast strategies at the plant level, and that trait divergence among genetic lineages is non-neutral. This supports the idea that plant populations evolve different slow-fast strategies along with different LES traits in order to adapt to contrasting climates.

We showed that LES trait correlations in *A. thaliana* follow the interspecific pattern^3,5^: individuals that invest a large amount of biomass per unit leaf area have a lower leaf assimilation rate and a longer leaf lifespan than plants that invest less biomass per unit leaf area. Moreover, the economics spectrum is still detected when scaling from leaf to whole-plant traits. This gives strong support to the idea that, at least in an herbaceous species such as *A. thaliana*, a trait value obtained on a single leaf using a standardized method, reflects the average phenotypic value expressed by all the leaves of an individual plant^61,62^. Furthermore, our results showed that the ranking of genotypes was similar along the leaf economics spectrum and the slow-fast continuum, suggesting that carbon economy at the leaf level is connected to the slow-fast strategies at the plant level. Up to now, functional ecology has favored interspecific comparisons, focusing on species trait means^63^, with the perspective of generalization and prediction of the whole biota functioning^64^. Conversely, model species and annual ones in particular, have often been considered as extreme and atypical strategies when examining the phenotypic space of the worldwide flora^11^. Here we showed how the eco-physiological examination of these species enrich our interpretation of interspecific trait covariations. Our results thus encourage future studies analyzing intraspecific trait covariations using model species such as *A. thaliana* in plant functional ecology to further advance our understanding of their underlying origins and mechanisms. Nonetheless, it is also true that our findings can be specific to the relatively simple organization and functioning of an annual rosette species. In particular, the correlations between leaf-level and whole-plant traits are presumably strongly variable among species. This relation is notably expected to be weaker in woody species because of the varying proportion of non-photosynthesizing tissues^65^. This result has a consequence for mass-based traits: when a given leaf trait increases by one unit, the same trait at the plant level increases by less than one unit. Our results illustrate this statement: leaf *A*_mass_ correlated with plant *A*mass with a slope below of 1. Similarly, leaf lifespan varied less across genotypes than plant age at maturity. As a result, there is a room for a decoupling between leaf and plant life history, even though the ranking among genotypes is globally conserved at the two organizational scales. Further explorations of how much leaf-level trade-offs and plant functioning are coordinated in herbaceous and woody species are needed.

Despite the autogamous nature of *A. thaliana* which makes it peculiar in eco-evolutionary studies, its large geographic distribution allows to explore multiple trait-environment relationships at large scales. These relationships are still hardly known and quantified for most functional traits and in most biomes^12,64^ because of the myriad of species-specific responses that can blur the general interspecific trends. Again, our results illustrate how intraspecific comparisons can help elucidating the mechanisms underlying these relationships, notable putative adaptive ones. Using the Q_ST_-F_ST_ comparisons, we brought evidence that *A. thaliana* regional populations are structured according to slow-fast traits, although better characterization of the genetic determinism of these phenotypes is needed. We reinforced this assertion identifying climatic factors related to this structure. The predicted distribution of slow-fast strategies across Europe revealed differential selection between roughly Norway, Sweden and Spain on one side, and central and Western Europe on the other. Selection for slow genotypes toward higher latitude in *A. thaliana*, specifically in North Swedish genotypes, is supported by previous findings on flowering time^66,67^. More surprisingly however, our results suggest that similar trait combinations representative of slow strategies are selected in two contrasted climates: Spain and Scandinavia, which are at the opposite edges of the *A. thaliana* latitudinal range. This clustering of *A. thaliana* genotypes echoes a recent study showing fixation of drought-related alleles in both Scandinavian and Spanish *A. thaliana* populations^68^. If we consider together the absence of significant effect of the kinship matrix on trait-trait relationships tested, the globally low average differentiation among genetic groups (F_ST_ = 0.11), and the phenotypic similarity observed at two distant locations, our results suggest that the genetic determinism of slow strategies as well as phenotypic differentiation could have occurred by convergence through adaptive processes. Thus, slow strategies could be selected in response to environmental stress in regions from nonetheless very different climates: low average temperature at Scandinavian sites and Mediterranean climate at Spanish sites. Interspecific studies at global scale revealed a negative relationship between conservative strategies and rainfall^3,69^, possibly linked to a higher investment in cell wall complex macromolecules to face drought stress^70^. Large-scale interspecific studies also reported a bias toward acquisitive strategies with increasing temperature in herbaceous species^71,72^. Similarly, theoretical frameworks suggest that drought and cold favor slow growing individuals in environments limited by water or temperature^73^. Together, this suggests a general selection pressure for slow strategies in stressful environments, as opposed to selection for fast strategies in non-stressing environments^23^. A promising avenue for future studies is to perform reciprocal transplant experiments to test the effect of drought and high temperatures on *A. thaliana* genotypes distributed along the slow-fast continuum.

Overall, our results suggest that slow-fast strategies are differentially selected in contrasted climates. Despite strong coordination of strategies from leaf level to plant level, slow-fast traits were significantly differentiated across populations and were associated with climatic conditions at the collecting sites, while significance was lacking for LES traits. By contrast, previous experiments under controlled conditions reported significant effects of temperature and soil water content on both whole plant and leaf traits in *A. thaliana*^74,75^. Complex interactions between climatic variables could change their relationships with phenotype in natural environments. This echoes the long standing search for global pattern of covariation between climate and LES traits in functional ecology and biogeography^3,71,76^. In addition to the expected anisometric relationship between plant and leaf traits (slope ≠1), room is left for leaf level traits to desynchronize from individual strategy. This suggests a potential for leaf traits to adapt to microclimatic conditions, including those at the canopy level. More broadly, our results corroborate the weak predictive power of leaf-level traits only in functional ecology when searching for adaption to particular climatic variable combination^12,13^. Indeed, even for an annual herb such as *A. thaliana*, whole-plant traits are more likely to capture adaptation to the environment compared to organ-level traits.

Using a model species, with large collections of well-characterized genetic material, appears particularly successful to go deeper into the evolutionary underpinning of major eco-physiological trade-offs, such as the LES and the slow-fast continuum. Combined with global climatic data, our findings notably revealed that adaptation to cold or dry habitats tends to favor slow strategies in *A. thaliana*. Next steps will be to merge approaches, and fully benefit from what a model species can provide both to genetics and ecophysiology. For instance, the climatic cues detected here despite the lack of climate data precision, is encouraging for the future of functional biogeography^64^. There is also evidence that the connection between functional trait and environmental adaptation requires a better characterization of plant fitness through demographic measures^26^. Comparative studies integrating demographic approach at population level are promising to understand how selection and macro-ecological gradients shape the evolutionary responses of plants to climate variation^24,26^.

## Materials and Methods

### Plant material

We used a total of 378 natural genotypes of *A. thaliana* L. Heynh sampled from the worldwide lines of the RegMap population (http://bergelson.uchicago.edu/wp-content/uploads/2015/04/Justins-360-lines.xls), which were genotyped for 250 K bi-allelic SNPs^44^. Seeds used in the present study were originally collected and multiplied by Fabrice Roux (UMR LIPM, Toulouse, France) and his collaborators.

### Growth conditions

Phenotype characterization was performed under controlled conditions in the high-throughput PHENOPSIS phenotyping platform^77^ to track daily growth. Seeds were kept in the dark at 4 °C for at least one week before sowing. Four to six seeds per genotype were sown at the soil surface in 225 ml pots filled with a 1:1 (v:v) mixture of loamy soil and organic compost (Neuhaus N2). The soil surface was moistened with one-tenth strength Hoagland solution, and pots were kept in the dark during 48 h under controlled environmental conditions (20 °C, 70% air relative humidity). Then, pots were placed in the PHENOPSIS growth chamber at 20 °C, 12 h photoperiod, 70% relative humidity, 175 µmol m^−2^ s^−1^ PPFD. Pots were sprayed with deionized water three times per day until germination, and then soil water content was adjusted to 0.35 g H_2_O g^−1^ dry soil (−0.07 MPa soil water potential) to ensure optimal growth^74,78^. After emergence of the fourth leaf, one individual plant was left in each pot.

### Measurements of plant traits

In order to standardize measurements for all genotypes, all traits were quantified when flower buds were macroscopically visible (*i.e*. bolting stage), and leaf traits were measured on the last adult leaf, fully exposed to light.

Net photosynthetic rate, relative expansion rate, lifespan, vegetative dry weight, as well as leaf area were determined for the leaf and the plant canopy. Net photosynthetic rate was measured at leaf (leaf *A*, nmol CO_2_ s^–1^) and whole-plant levels (plant *A*, nmol CO_2_ s^–1^) under growing conditions using, respectively, the leaf cuvette provided with the infrared gas analyzer system (CIRAS 2, PP systems, USA), and a whole-plant chamber prototype designed for *A. thaliana* by M. Dauzat (INRA, Montpellier, France) and K. J. Parkinson (PP System, UK) (see^28^). Leaf and whole-plant photosynthetic rates were both expressed on dry mass basis (leaf *A*_mass_ and plant *A*_mass_, nmol CO_2_ g^–1^ s^–1^). Due to time constraints, we measured photosynthetic rates for 319 and 348 genotypes at the leaf and whole-plant levels (306 in common), respectively. We estimated the age of maturity by the number of days from germination to the appearance of the flower bud. Then, plants were harvested, and individual fresh weight was determined. The leaf used for photosynthetic measurements was identified and processed separately, and detached rosettes were kept in deionized water at 4 °C for 24 h, and water-saturated weight was determined. Individual leaves were then attached to a sheet of paper and scanned for subsequent determination of the leaf number and total leaf area using ImageJ^79^. Dry weight of laminas and petioles were obtained after drying for 72 h at 65 °C. Rosette dry weight was expressed as the sum of lamina and petiole dry weights. Leaf mass per area was both calculated for the leaf used for photosynthetic measurements (LMA, g m^−2^) and for the whole-rosette (plant LMA, g m^−2^) as the ratio of lamina dry mass to lamina area. Relative growth rate (RGR, mm² mm^−2^ d^−1^) and leaf lifespan (LLS, d) were estimated from automated daily pictures of the rosettes. More precisely, a sigmoid curve was fitted to rosette area as a function of time in order to extract growth parameters, where RGR was calculated as the slope at the inflection point^80–82^. Using daily pictures, we tracked three consecutive leaves from birth (emergence) to death (full senescence). For each plant, leaf duration was calculated as the average number of days from leaf emergence to senescence.

### F_ST_ and P_ST_ estimates

In order to perform population genetic analyses, genetic groups were identified by genetic clustering of 378 genotypes, using the 250 K SNPs data available from Horton *et al*.^44^. Clustering was performed with ADMIXTURE^83^ after linkage disequilibrium pruning (*r*^2^ < 0.1 in a 50 kb window with a step size of 50 SNPs) with PLINK^84^, resulting in 24,562 independent SNPs used for subsequent analyses. A cross-validation for different numbers of clusters (k = 1 to k = 10) showed that the set of studied genotypes can be separated into five groups representative of different genetic lineages (cross validation error = 0.89). Following the same approach as the 1001 genomes project^45^, we assigned each genotype to a group if more than 50% of its genome derived from the corresponding cluster. The 74 genotypes not matching this criterion were labelled “Admixed” and were not used for the F_ST_ and P_ST_ calculation. The groups genetically defined were also geographically distinct as shown by early studies^44^. We measured the genetic population structure using Weir and Cockerham F_ST_ statistic for all the 24,562 SNPs, as well as mean F_ST_ genome-wide. To determine the neutral F_ST_ value, we calculated the median of the significant F_ST_ values of the intergenic SNPs. More specifically, we filtered the SNPs dataset keeping the intergenic SNPs only, assuming that they are rather prone to endure neutral differentiation processes. We then calculated a F_ST_ value for each intergenic SNP with the attribution of population described above. To test for the significance of the F_ST_ values, we randomized 1000 times the population attribution to the genotypes in order to provide a null distribution of F_ST_. F_ST_ values higher than the 95^th^ quantile of their null distribution were stated as significant. The phenotypic population structure is ideally evaluated using Q_ST_, an analogue of F_ST_ measure^85,86^. We estimated Q_ST_ using a phenotype-based surrogate (P_ST_)^86^ which depends on among-population and overall heritability of the trait (c and *h*², respectively) computed as follow:

$${P}_{ST}=\frac{c\,{\sigma }_{B}^{2}}{c\,{\sigma }_{B}^{2}+{h}^{2}{\sigma }_{W}^{2}}$$

where B and W are the between- and within-population part of variance, respectively. Note that within-population variance is not multiplied by 2 since *A. thaliana* is mainly autogamous and homozygote at all loci. Under the null assumption (H0: c = *h*² = 1), P_ST_ and Q_ST_ are analogous. Sensitivity analysis consists in calculating the P_ST_ value and its 95% confidence interval for a gradient of hypothetical c and a given *h*². P_ST_ is a robust estimator of Q_ST_ when the critical value of c/*h*² is low, i.e. the minimum value of c/*h*² for which the lower limit of P_ST_ 95% CI is higher than the neutral F_ST_. Then, a value of Q_ST_ higher than neutral F_ST_ means that the phenotypic differentiation between populations is larger than expected by demographic events alone, in particular genetic drift, and is thus indicative of diversifying selection on traits^41,87^. We used parametric bootstrap method to generate 95% CI around P_ST_ values with the package MCMCglmm in R (10,000 iterations).

### Statistical analysis

Climate variables at the sampling sites of each genotype were extracted from the CHELSA database (http://www.chelsa-climate.org/), with a 2.5 arc-minutes resolution. The effect of climatic variables on traits was tested using linear model regressions. All analyses were performed in R 3.4.1 (R Core Team, 2017). Whittaker’s biomes were plotted using the BIOMEplot function provided by G. Kunstler (https://rdrr.io/github/kunstler/BIOMEplot/src/R/biomes-plot.R). All leaf and plant traits, but RGR, were log_10_ transformed when Gaussian distribution is required for statistical analyses. Principal component analysis (PCA) was performed using the package FactoMineR. The package nlme was used to perform linear models and phylogenetic generalized least squares regressions. We performed phylogenetic regressions including a relatedness matrix as covariance matrix, obtained after running the PLINK–make-rel command across the 250 K SNPs from the RegMap data. SMA regressions between leaf and plant traits were performed using the package SMATR^88^, and phylogenetic SMA regressions using the Phyl.RMA function of the Phytools package. The phylogenetic tree required for SMA regression has been produced with Tassel using the RegMap SNPs data^44^.

## Supplementary information

Supplementary Figures and table

## Data Availability

Data are deposited in the Dataverse repository: 10.15454/B3W0OS.
