# Supplementary material for: Leaf economics and slow-fast adaptation across the geographic range of Arabidopsis thaliana
Source: Sci Rep. 2019 Jul 24;9:10758. doi: 10.1038/s41598-019-46878-2 (PMC6656729; doi:10.1038/s41598-019-46878-2)

Leaf economics and slow-fast adaptation across the geographic range of *Arabidopsis thaliana*

Kevin SARTORI [+,*,1], François VASSEUR[*,1,2], Cyrille VIOLLE[1], Etienne BARON[1], Marianne GERARD[1], Nick ROWE[3], Oscar AYALA-GARAY[2,4], Ananda CHRISTOPHE[1], Laura GARCIA DE JALÓN[1], Diane MASCLEF[2], Erwan HARSCOUET[1], Maria DEL REY GRANADO[1], Agathe CHASSAGNEUX[1,5], Elena KAZAKOU[1,6] and Denis VILE[2]

Supplemental Information

Figure S1: Leaf economics spectrum is consistent from leaf to plant level as a result of strong trait covariation from leaf to plant level in *A. thaliana*. All traits were log10 transformed. Principal component analysis of LES traits (Amass, Assimilation rate; LLS, leaf lifespan; LMA, leaf mass per area): correlation circle of variable at the leaf (a) and plant levels (b), and individuals at the leaf (c) and plant (d) levels. Covariation between leaf and plant traits: Leaf mass per area (e), Assimilation rate (f), life history (g) and principal components (h). Dashed lines represent the identity relation and continuous lines represent standard major axis regression when significant.

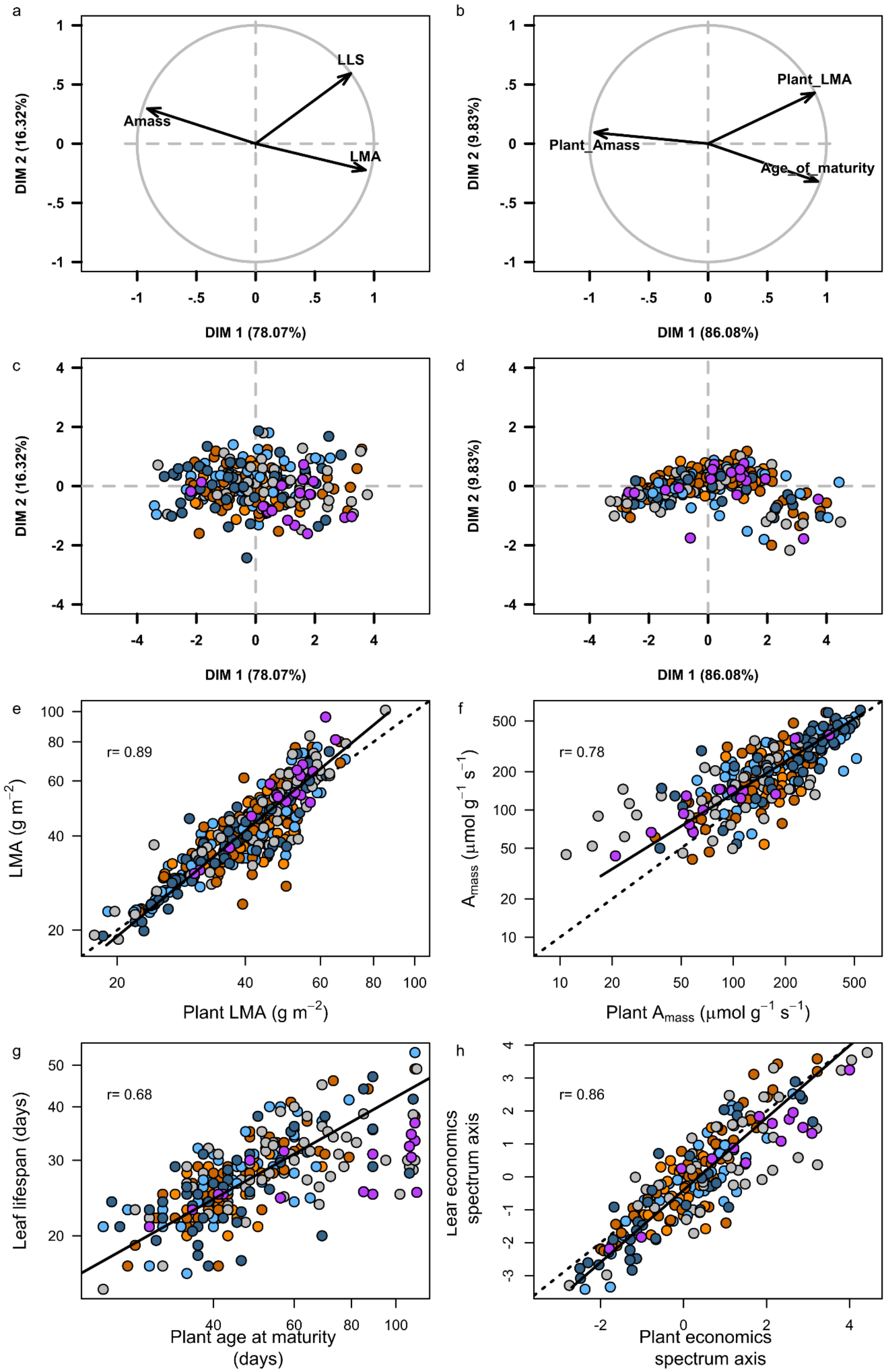

a
LLS
Amass
LMA
DIM 2 (16.32%)
DIM 1 (78.07%)
b
Plant_LMA
Plant_Amass
Age_of_maturity
DIM 2 (9.83%)
DIM 1 (86.08%)
c
DIM 2 (16.32%)
DIM 1 (78.07%)
d
DIM 2 (9.83%)
DIM 1 (86.08%)
e
r= 0.89
LMA (g m^-2)
Plant LMA (g m^-2)
f
r= 0.78
A_mass (µmol g^-1 s^-1)
Plant A_mass (µmol g^-1 s^-1)
g
r= 0.68
Leaf lifespan (days)
Plant age at maturity (days)
h
r= 0.86
Leaf economics spectrum axis
Plant economics spectrum axis

Figure S2: Influence of the c/*h*² ratio on the significance and robustness of the P_{ST}. Phenotypic differentiation (PST, black vertical lines) relatively to the distribution of genetic differentiation (FST), its median (red dashed line), 90th quantile (ligth blue) and 95th quantile (dark blue) (a). Sensitivity analysis: the red line indicates the F_{ST} median value, the black line indicates the PST values and the grey shape represents its 95% confidence interval as a function of the c/*h*² ratio. The phenotypic differentiation is significant and its estimator (P_{ST}) robust when PST confidence interval and FST overlap at low c/h² values. Relative growth rate (mm^2 mm-2 d^{-1}) (b), the age at maturity (days) (c), plant assimilation rate (nmol CO2 g^{-1} s^{-1}) (e), plant leaf mass per area (g m^{-2}) (f) and position on the slow-fast continuum (f).

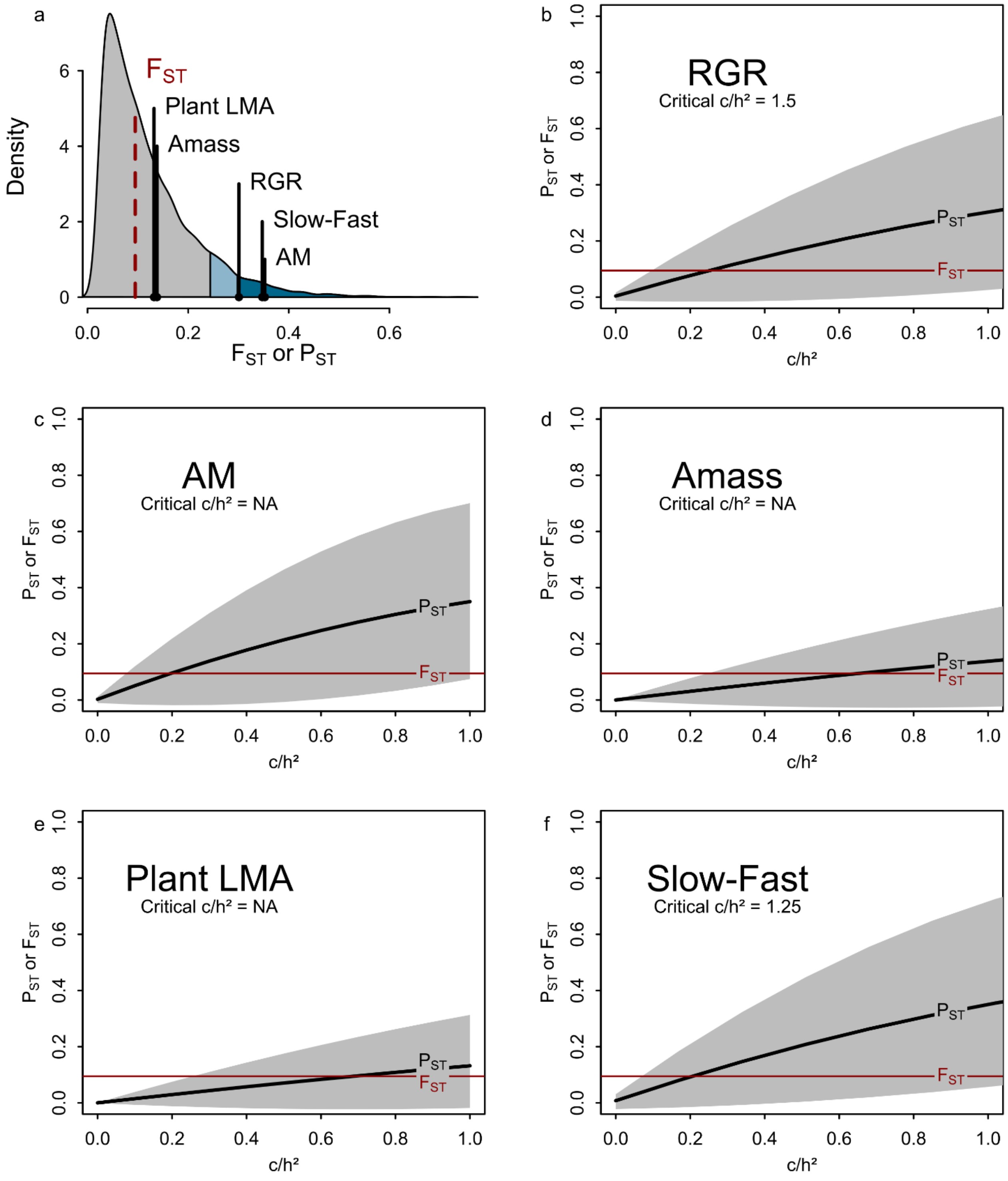

Figure S3: Influence of the c/*h*² ratio on the significance and robustness of the P_{ST}. Phenotypic differentiation (PST, black vertical lines) relatively to the distribution of genetic differentiation (FST), its median (red dashed line), 90th quantile (ligth blue) and 95th quantile (dark blue) (a). Sensitivity analysis: the red line indicates the FST median value, the black line indicates the PST values and the grey shape represents its 95% confidence interval as a function of the c/h^2 ratio. The phenotypic differentiation is significant and its estimator (PST) robust when PST confidence interval and FST overlap at low c/h^2 values. Leaf lifespan (days) (b), leaf assimilation rate (nmol CO2 g–1 s–1) (c), leaf mass per area (g m-2) (d) and position on the leaf economics spectrum (e).

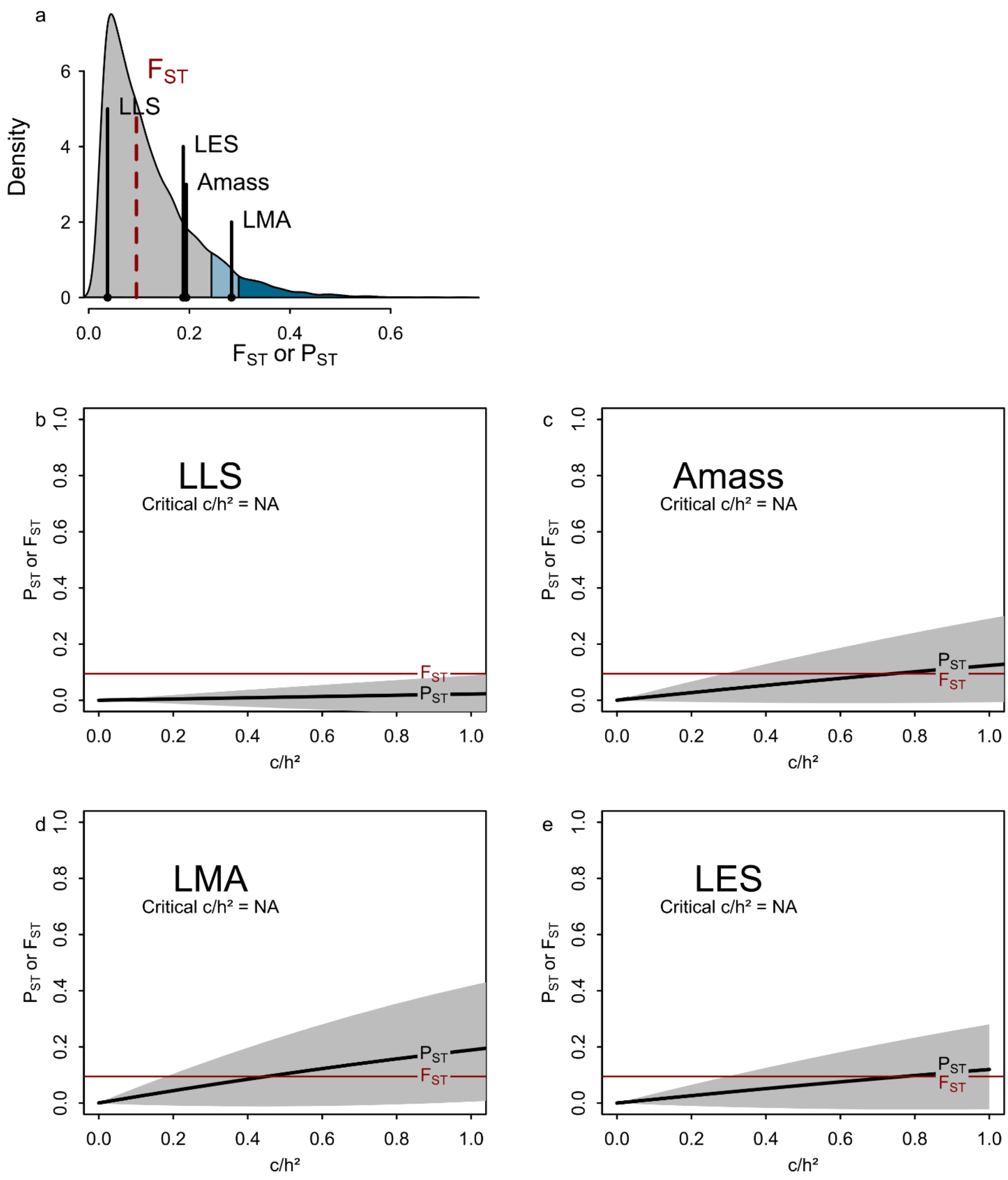

Table S1: Correlation coefficient between plant traits and climatic variables at the collecting sites. All traits but RGR were log10 transformed. Significance code: ***; p<0.001, **; p<0.01, *; p<0.05, ns; non-significant. *Abbreviations: RGR; Relative Growth Rate, AM; Age at maturity, Amass; mass based assimilation rate, LMA; Leaf Mass per Area, LLS; leaf lifespan, SFC; slow-fast continuum, LES; leaf economics spectrum, BIO1; Annual Mean Temperature, BIO2; Mean Diurnal Range, BIO3; Isothermality, BIO4; Temperature Seasonality, BIO5; Max Temperature of Warmest Month, BIO6; Min Temperature of Coldest Month, BIO7; Temperature Annual Range, BIO8; Mean Temperature of Wettest Quarter, BIO9; Mean Temperature of Driest Quarter, BIO10; Mean Temperature of Warmest Quarter, BIO11; Mean Temperature of Coldest Quarter, BIO12; Annual Precipitation, BIO13; Precipitation of Wettest Month, BIO14; Precipitation of Driest Month, BIO15; Precipitation Seasonality, BIO16; Precipitation of Wettest Quarter, BIO17; Precipitation of Driest Quarter, BIO18; Precipitation of Warmest Quarter; BIO19; Precipitation of Coldest Quarter*

	RGR	AM	Amass	LMA	LLS	SFC	LES
BIO1	0.34 ***	-0.22 ***	ns	-0.13 *	ns	-0.18 **	-0.13 *
BIO2	0.12 *	-0.17 **	ns	ns	ns	-0.16 **	ns
BIO3	0.23 ***	-0.29 ***	ns	-0.14 *	ns	-0.31 ***	-0.2 **
BIO4	-0.16 **	0.12 *	-0.15 *	0.14 **	ns	0.15 *	0.14 *
BIO5	0.2 ***	-0.14 *	ns	ns	ns	ns	ns
BIO6	0.28 ***	-0.17 **	ns	-0.14 *	ns	-0.16 **	ns
BIO7	ns	ns	-0.13 *	ns	ns	ns	ns
BIO8	ns	ns	ns	ns	ns	ns	ns
BIO9	ns	ns	ns	ns	ns	ns	ns
BIO10	0.25 ***	-0.13 *	ns	ns	ns	ns	ns
BIO11	0.3 ***	-0.2 ***	ns	-0.14 *	ns	-0.18 **	-0.14 *
BIO12	0.15 **	-0.16 **	ns	-0.12 *	-0.16 **	-0.16 **	-0.14 *
BIO13	ns	-0.14 **	0.12 *	-0.15 **	-0.13 *	-0.14 *	-0.14 *
BIO14	0.16 **	-0.16 **	ns	ns	ns	-0.15 *	ns
BIO15	-0.11 *	ns	ns	ns	ns	ns	ns
BIO16	ns	-0.15 **	0.13 *	-0.16 **	-0.14 *	-0.15 *	-0.15 *
BIO17	0.16 **	-0.16 **	ns	ns	ns	-0.15 *	ns
BIO18	ns	-0.11 *	ns	ns	-0.21 ***	ns	ns
BIO19	0.19 ***	-0.15 **	ns	-0.11 *	ns	-0.14 *	ns

Figure S4: Mean trait comparison between genetic groups. Leaf (a) and plant (b) assimilation rate per unit mass (Amass), leaf (c) and plant (d) life history trait, leaf mass per area (LMA) (e), plant level LMA (f). F statistics and letters are given from Tukey tests, all traits were log-transformed.

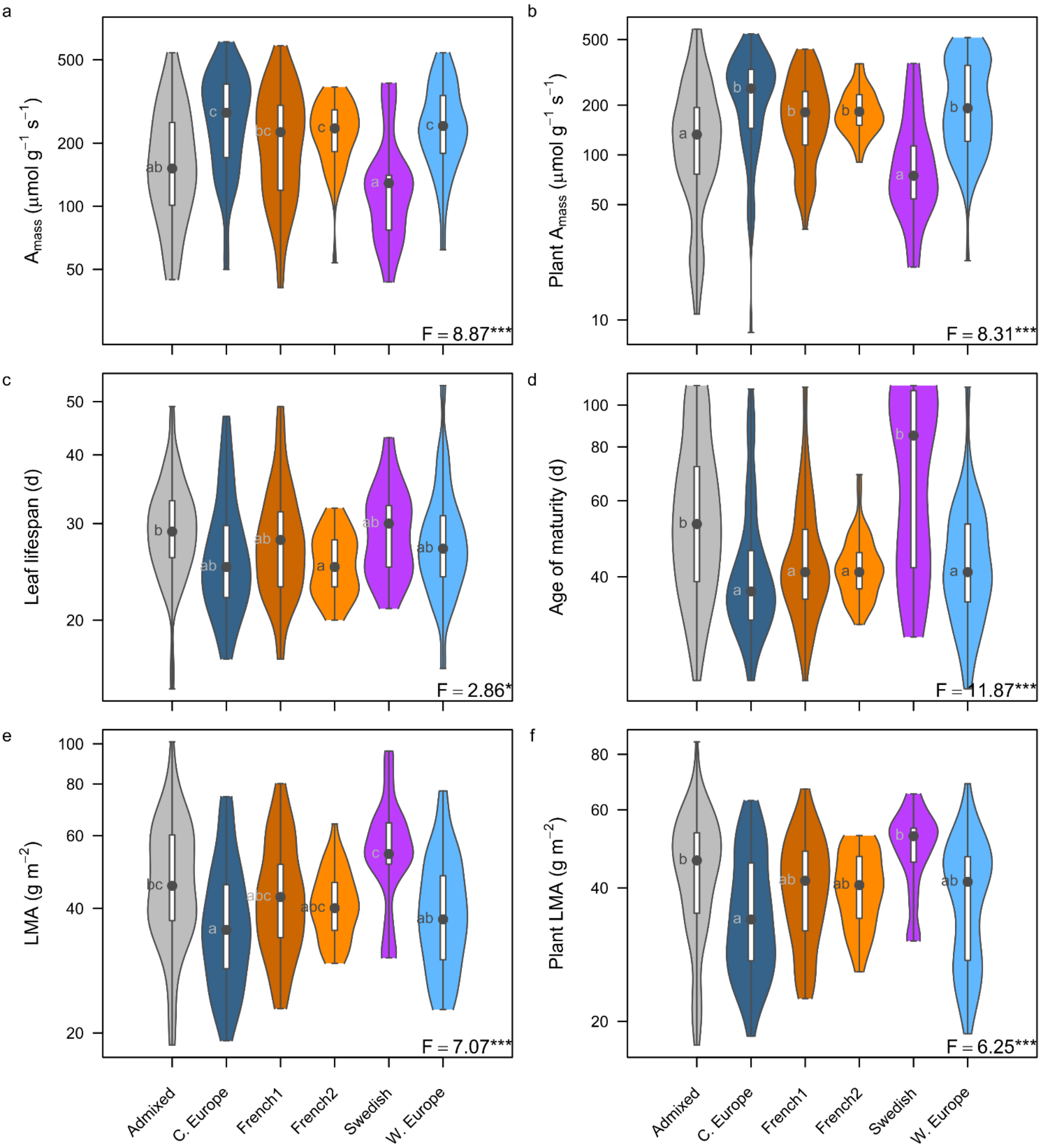

Supplement: Supplementary file 1 — Supplementary Figures and table [file 41598_2019_46878_MOESM1_ESM.pdf]
